# Application of ICP-MS, INAA and RNAA to the determination of some “difficult” elements in infant formulas

**DOI:** 10.1007/s10967-016-5042-8

**Published:** 2016-09-20

**Authors:** Ewelina Chajduk, Halina Polkowska-Motrenko

**Affiliations:** Laboratory of Nuclear Analytical Techniques, Institute of Nuclear Chemistry and Technology, 16 Dorodna, 03-195 Warsaw, Poland

**Keywords:** Trace elements, Powdered milk, Grain porridges, Neutron activation analysis, Inductively coupled plasma mass spectrometry

## Abstract

In this work a determination of selected elements in the infant formulas commercially available on the Polish market was done. 14 different materials (milk-based formulas and grain porridges) were analyzed. Both, inductively coupled plasma mass spectrometry (ICP-MS) and instrumental neutron activation analysis (INAA) were applied for the determination of As, Cr, Fe and Se, which are recognized as the problematic elements for ICP-MS. For As and Se, the radiochemical NAA was also used. The daily intake of Se and Fe in the age 0–6 months for non-breast fed infants was estimated and compared with present safety limits.

## Introduction

Good nutrition is essential for the growth and development that occur during an infant’s first months of life. During infancy, a period of rapid growth, nutrient requirements are higher than at any other time in the life cycle. Breast milk is strongly suggested as the only source of nutrition for the first 6 months of life. However, data show that only 3 % of European babies are breast fed [1]. Therefore, milk-based formulas and/or milk substitutes have to be used when it is difficult to bring up an infant on mother’s milk. A number of commercial infant food ready to feed are available, intended for the consumption before the age of 6 months. According to EC Commission Directive (2006/125/EC), the nature of these products require nutritional labeling showing principal nutrients they contain [2]. Depending on producers of the infant formulas and food, data on the concentration of the selected essential elements are given. However, most essential trace elements in infant formula have received very little attention. Increasing environmental pollution, as well as the process of food preparation technology, can raise contamination with heavy metals. For example, the rice-based infant food contains significantly higher amounts of As. It was recently found, that the consumption of powdered milk with high As content caused pancreatic cancer [3]. Therefore, the toxic elements should be also determined in these products [1–3]. The necessity of trace elements determination in infant formulas resulted in numerous publications [2–10]. Most of them concerns the determination of selected elements in milk replacement formulas based on cow’s milk and presents comparison between breast milk and investigated samples. Most often, for the determination of elements at trace and ultratrace level, inductively coupled plasma mass spectrometry [1, 4, 5, 7], inductively coupled plasma optical emission spectrometry (ICP-OES) [5, 6], electrothermal atomic absorption spectrometry (ET-AAS) [7, 9] are used. As baby food products contain very small amounts of trace elements, the analytical methods used for their determination must be characterized by low limits of detection. The published data show, that the determination of macro- and micronutrients in milk is carried out in many countries. The interesting data are presented by Pandelova and co-workers [10]. During these studies, Ca, Cd, Cu, Fe, Hg, Mn, Ni, Pb, Se and Zn were determined in the most consumed baby foods in six European countries including infant formulas, solid foods and beverages. Determination of 26 elements in infant formulas coming from USA, UK and Nigeria by ICP-OES was presented [11]. It has been shown that there were significant variations of some of the element levels across the infant formula brands. Polish commercially available infant formulas were investigated in order to find mineral composition, lactose and protein content [6]. In these studies, no toxic elements such as As and Cr have been determined, similarly, essential elements content like Se and I have not been taken into consideration. Mineral composition (Mg, Zn, Cu, Ca, Mn, Na, Fe, K) in powdered milk was investigated by Winiarska-Mieczan [12]. All elements were determined by flame atomic absorption spectrometry (F-AAS). In comparison to recommended norms, all examined milk samples contained much more Ca and Cu, whereas Mg and Mn were deficient. All presented data confirm differences between human milk and infant formulas. Also differences between infant formulas are observed, what could be attributed to the different manufacturing practices, variations in quality of raw materials, finished products and packaging containers used by infant formula manufacturers. For this reason it is necessary to continue these studies in order to prepare new infant formulas and infant food containing the macro- and micronutrients in levels more similar to human milk. The position of NAA among the analytical techniques is still strong despite some weaknesses like the necessity of having access to a nuclear reactor, radiochemical laboratory, long analysis time, etc. and NAA is, with no doubt, a valuable method in inorganic trace analysis, especially important in certification of reference materials [13, 14]. During recent years, it has been proved that instrumental NAA (INAA), because of its unique possibilities, can meet Consultative Committee for Amount of Substance (CCQM) criteria for a primary method of measurement (PMM) [15]. Another important feature of INAA is that it is free from the problems connected with digestion of difficult matrices and potential contamination of a sample from reagents. Moreover, by changing irradiation and cooling parameters as well as measurement time, the effect of the interfering radionuclides on the results of analysis of particular elements can be decreased [16]. In the case of the determinations of As, Cr, Fe and Se by ICP-MS [17], there is a number of spectral interferences, which may influence the measurement, particularly for very low content. So, ICP-MS as analysis method for these elements possesses some limitations. In the case of NAA—all of the interested elements can be determined directly or after radiochemical separation.

The aim of this study was the critical evaluation of analytical performance of ICP-MS and INAA for the determination of As, Cr, Fe and Se in infant formulas. The complementary use of NAA and ICP-MS to study the elemental composition of the food samples, allows to obtain reliable and richer analytical information. In the case of As and Se determinations, ratio primary reference measurement procedures (RPRMPs) based on radiochemical NAA, as the methods of the highest metrological quality, have been used to confirm the accuracy of the obtained results. The daily intake of selected elements in the age 0–6 months for non-breast fed infants was estimated and compared with present safety limits.

## Experimental

### Reagents and materials

In the preparation of all solutions, 18 MΩ cm grade water from Milli-ORG Millipore Co. purification system was used. For digestion process 65 % HNO_3_ (p.a. Sigma, purified by sub-boiling point distillation), 40 % HF (suprapure, MERCK), H_2_O_2_ (p.a., FLUKA) were applied. Standards used for ICP-MS and NAA were supplied by Perkin Elmer as stock standard solutions of 100 µg mL^−1^ and 1 mg mL^−1^. Working mixed standard solutions (2–200 ng mL^−1^) were prepared by dilution of the stock standard solutions with 0.7 % nitric acid.

#### For RNAA

Reagents and materials used for preparation of column filling are described in details previously [18].

The following commercially available materials have been tested: 4 milk-based formulas; 2 hypoallergenic infant formulas; milk-, soya-, and gluten free (dietary food), produced from rice and carob germs; 1 milk free, soya based formula; 3 rice-based porridges; oatmeal porridge; buckwheat porridge; millet porridge; spelt porridge.

Certified reference materials (CRMs) were used for the validation of method: non-fat milk powder 1549 (NBS), soya bean flour INCT-SBF-4 (INCT), rye flour IAEA-V-8 (IAEA), Polish virginia tobacco leaves PVTL-6 (INCT).

### Apparatus

To prepare samples, CRMs and standards, analytical and micro-analytical balances Sartorius MC5 and Sartorius BP221S, calibrated using national mass standards traceable to the international standards were used. The microwave digestion system (Anton Paar Multiwave 3000, USA) equipped with temperature and pressure regulation was used for digestion of the samples. ICP-MS instrument ELAN DRC II (Perkin Elmer) with crossflow nebulizer with Scott double-pass spray chamber and Ni cones was used. To improve the detection limits for ICP-MS, the ultrasonic nebulizer U6000AT + (Cetac) was used.

To perform gamma-ray spectroscopic measurements the following detectors were used:

- 255 cm^3^ HPGe well-type detector (Canberra) with associated electronics (resolution 2.15 keV for 1332 keV ^60^Co line, efficiency ca thermal neutron fluxa. 55 %), coupled to a multichannel analyzer and spectroscopy software Genie-2000 (Canberra).
- 180 cm^3^ HPGe well-type (Canberra) with associated electronics (resolution 2.09 keV for 1332 keV ^60^Co line, efficiency ca. 30 %), coupled to a multichannel analyzer TUKAN (The Andrzej Soltan Institute for Nuclear Studies, Świerk, Poland).

### Analytical procedures

#### ICP-MS

Microwave digestion of sample mass ~250 mg with HNO_3_, H_2_O_2_ and HF (6:2:1) was applied. Obtained solutions were diluted with 0.7 % HNO_3_ and In was added as an internal standard prior to analysis. The isotopes selected for measurement were as follow: ^75^As, ^52^Cr, ^57^Fe, ^78,80,82^Se, since they are free from interference and are sufficiently abundant. The applied instrument operation conditions are summarized in Table 1. Indium-115 was selected as an internal standard, due to its absence in the analyzed samples.

**Table 1:** Operation parameters for ICP-MS analysis

Mass spectrometer ELAN DRC II	RF power: 1000 W Plasma gas flow: 13.0 L min^−1^ Auxiliary gas flow: 1.2 L min^−1^ Nebulizer gas flow: 0.92 L min^−1^ Lens voltage: 6.75 V Detector mode: Dual Analog detector voltage: −1650 V Pulse detector voltage: 800 V Working mode: standard, DRC Cones: Ni

#### INAA

The package containing samples sandwiched between standards, certified reference materials (CRMs) and blanks, was irradiated in the nuclear reactor MARIA, for 50 min at a thermal neutron flux of 10^14^ cm^−2^ s^−1^. The irradiation, cooling and counting times were chosen according to half-lives of the determined elements. Table 2 presents irradiation and measurements details.

**Table 2:** Irradiation and measurement conditions

Nuclide used	Irradiation time	Cooling time (days)	Measurement time (s)
^76^As	50 min	1–3	2000–5000
^51^Cr, ^59^Fe, ^75^Se		10–14	10,000–25,000
^*76*^ *As*-*RNAA*		*1*–*2*	*2000*–*3600*
^*75*^ *Se*-*RNAA*		*2*–*5*	*2000*–*5000*

The data in the table is highlighted in italics to distinguish instrumental and radiochemical NAA

#### RNAA for As and Se

The samples of biological material, elemental standards, CRMs and blank were placed in polyethylene (PE) containers, irradiated in the nuclear reactor MARIA, for 50 min at a thermal neutron flux of 10^14^ cm^−2^ s^−1^ and cooled for appropriate cooling time. Then, the samples were acid digested using a high-pressure microwave system. After decomposition, the samples were subjected to separation procedure. The details of the radiochemical procedures were published previously [19, 20]. The count rate of the separated radionuclides were measured by gamma-ray spectrometry.

## Results and discussion

### ICP-MS measurements

In the case of instrumental techniques, where is necessary to have sample in solution, sample digestion is a critical step in analysis. It is due to risk of contamination and analytes loss. In the case of analyzed milk based formulas, microwave digestion with HNO_3_ and H_2_O_2_ concentrated was sufficient; assured total dissolution of the samples to the clear solution. In the case of grain porridges; addition of HF was necessary, due to high content of silicon [21].

ICP-MS is suitable technique for the determination of trace elements in food samples due to its precision, low detection limit (LOD), wide linear dynamic range etc. However, some elements e.g. As, Cr, Fe and Se may be classified as the difficult elements for ICP-MS, mainly due to presence spectral and non-spectral interferences. Table 3 contains the most common spectral interferences. As can be seen, the polyatomic ions formed in the argon plasma causes most of them. Applying the dynamic reaction cell (DRC) mode could eliminate part of polyatomic interferences that have caused problems in the direct analysis of infant formulas. Arsenic has only one isotope (*m*/*z* 75), its measurement is often impossible in chloride-containing samples because of presence ^40^Ar^35^Cl^+^ or ^40^Ca^35^Cl^+^. In the case of Cr, its analysis is difficult due to presence of ^40^As^12^C at *m*/*z* 52. Iron determination is hampered, especially in calcium rich samples, due to interferences of different polyatomic ions produced by oxygen, argon and calcium on ^56^Fe (^40^Ar^16^O^+^ and ^40^Ca^16^O^+^) and ^57^Fe (^40^Ca^16^O^1^H^+^, ^40^Ar^16^O^1^H^+^). With conventional quadrupole ICP-MS, the most abundant isotopes of selenium (^80^Se and ^78^Se) cannot be used for the determination due to the interference of ^40^Ar^40^Ar^+^ and ^40^Ar^38^Ar^+^ ions. As a result, selenium is usually determined using the ^82^Se isotope (8.7 % abundance). It should be noted, that in the case of spectral interferences, the intensity of the interfering species depends on the composition of the sample and therefore varies significantly. The usage of DRC reduces the amount of the polyatomic interferences. By a numerous of different ion–molecule reaction mechanisms, the gas (NH_3_, CH_4_, O_2_, He and other) reacts with the interfering ions to form products that will not interfere with the determined ion.

**Table 3:** Polyatomic spectral interferences hampered accurate determination of As, Ce, Fe and Se by ICP-MS

Isotope	Interferences
^75^As	^40^Ar^35^Cl, ^40^Ca^35^Cl
^52^Cr	^40^As^12^C
^56^Fe	^40^Ar^16^O, ^40^Ca^16^O
^57^Fe	^40^Ca^16^O^1^H, ^40^Ar1^6^O^1^H
^74^Se	^36^Ar^38^Ar, ^37^Cl_2_, ^42^Ca^16^O_2_, ^40^Ca^16^O^18^O, ^74^Ge
^76^Se	^38^Ar_2_^+^, ^44^Ca^16^O_2_, ^36^Ar^40^Ca, ^76^Ge
^77^Se	^40^Ar^37^Cl, ^76^SeH
^78^Se	^40^Ar^38^Ar, ^38^Ar^40^Ca, ^77^SeH
^80^Se	^40^Ar_2_, ^40^Ar^40^Ca, ^79^BrH, ^32^S^16^O_3_, ^80^Kr
^82^Se	^40^Ar^42^Ca, ^81^BrH, ^34^S^16^O_3_, ^82^Kr

In present study, in the case of ICP-MS, in standard working mode, only determination of Fe was possible. The influence of spectral interferences on the determination of Fe was visible in all analyzed formulas. Due to high content of iron in food samples reliable results were obtained, although they were burdened with considerable uncertainty (U, k = 2) which was estimated at 20 %. Application of NH_3_ as reaction gas, allowed the determination of Cr in samples with chromium content above 100 ng/g, what was impossible in normal working mode. It also allowed to obtain results for Fe with lower uncertainty than without DRC. For arsenic, ammonia gas was not sufficient to obtain any results. Although ArCl^+^ reacts with NH_3_, CaCl^+^ is unreactive due to the high Ca–Cl bond strength. So, CaCl^+^ cannot be eliminated in this manner. As a result, the loss of As sensitivity is great, which would make trace-level measurements impossible.

In this study, two different samples introduction systems were applied: with crossflow nebulizer with Scott double-pass spray chamber and ultrasonic nebulizer. In the case of classic sample introduction system, practically only iron could be determined in this type of samples. When ultrasonic nebulizer was used; the signal intensity has increased about one order of magnitude in comparison with crossflow nebulizer, which enabled the determination of Cr and Se.

### NAA measurements

In the case of INAA, As activity was measured after 1–3 days of cooling, Cr and Fe- after 10–12 days. In the case of selenium due to Bremsstralung effect of β particles of ^32^P (T_1/2_ = 14.3 days), the cooling time was extended to 3 weeks. The direct determination of arsenic in cow-based milk was not possible, due to high activity of samples coming from sodium, potassium and phosphorus, mainly. In the case of chromium-52, the estimated uncertainty is relatively higher in comparison to Fe and Se, due to increased content of Cr in containers used for irradiation. Elaborated previously ratio primary reference measurement procedures by RNAA, have been used for the determination of Se and As in chosen infant formulas. Their use was proven in certification of reference materials and in checking the assigned values in proficiency tests [18, 22]. RPRMPs expanded uncertainties are of 2–3 % and are comparable to ID-MS methods. These procedures are the only methods of such high metrological quality which can be used for the determination of trace amounts of monoisotopic elements like arsenic. In the case of selenium, the application of radiochemical separation significantly reduces the cooling—c.a. 2 weeks.

A comparison of the results of As, Cr, Fe and Se determination in CRMs obtained by ICP-MS, INAA and RNAA is shown in Table 4. As can be seen, the results are in good agreement with the certified values. The described procedures give the accurate results. The main difference is in the value of uncertainties. In the case of ICP-MS, due to large number of isobaric interferences, small content of analytes and required sample dilution after microwave digestion, investigated elements could be determined with the expanded uncertainty U (k = 2) at the level of 15–20 %, whereas for INAA, U (k = 2) was estimated at 5–8 % for all elements. The detection limit of INAA method for rice-based samples and porridges are lower than for powdered milk. In the case of the determination of Fe in milk LOD is 0.66 µg g^−1^ and in rice formulas −0.45 µg g^−1^. Application of RPRMP based on RNAA allows to obtain results with lower LOD, lower uncertainty (2–3 %) and shorten time of analysis. For As in rice formulas, LOD is 32 ng g^−1^ for INAA and 7 ng g^−1^ for RNAA. LOD of Cr by ICP-MS method is 300 ng g^−1^ and 100 ng g^−1^ when DRC mode is applied. Takin the above into account, the application of INAA method can be considered the best solution for the determination of Cr, Fe and Se when the concentration of these elements are low. Determination of As below 100 ng g^−1^ is possible by NAA and above this concentration level also by ICP-MS method.

**Table 4:** Analysis of certified reference materials

Non-Fat Milk Powder 1549 (NBS)				
	ICP-MS	INAA	RNAA	Certified values
Cr, ng g^−1^				2.6 ± 0.7
Fe, µg g^−1^		2.04 ± 0.20		1.78 ± 0.10
Se, µg g^−1^	0.15 ± 0.03	0.11 ± 0.01	0.106 ± 0.004	0.11 ± 0.01

Soya Bean Flour INCT-SBF-4 (INCT)				
	ICP-MS	INAA	RNAA	Certified values
Cr, ng g^−1^		280 ± 35		(230)
Fe, µg g^−1^	88.0 ± 6.5	100.5 ± 15.0		90.8 ± 4.0

Polish Virginia Tobacco Leaves INCT-PVTL-6				
	ICP-MS	INAA	RNAA	Certified values
As, ng g^−1^	105 ± 15		133 ± 4	138 ± 10
Cr, ng g^−1^	982 ± 147	1095 ± 88		(911)

(…) information values

### Characterization of analyzed samples

Table 5 contains the obtained results of analysis of chosen infant formulas. Se and As have been determined by RNAA, Fe by INAA, and Cr by ICP-MS in DRC mode. The concentration of the determined elements in cow based milk is similar, independently of the producer and added ingredients (nucleotides, prebiotics etc.). Fe content varied between 46.1 and 71.7 µg g^−1^. In the case of selenium, its content was similar in all samples and amounted to 0.12–0.20 µg g^−1^. Chromium was below detection limit of ICP-MS method, which is relatively high (100 ng g^−1^). In these samples As was not determined by NAA because of very high radioactivity of ^24^Na. In soya milk, selenium and iron content was similar as in cow-based milk, arsenic content was 50.6 ng g^−1^, the concentration of chromium was at the level of 100 ng g^−1^. The major differences in the composition were observed for porridges and hypoallergenic infant formulas. Arsenic was present in almost all samples; only in buckwheat porridge it was below 7 ng g^−1^. Its content varied in the range of 13.8–72.2 ng g^−1^. The highest concentration was observed for hypoallergenic formula (rice and carob germs). Selenium was present at the level below 21.4 ng g^−1^. The concentration of chromium was at the level below 100 ng g^−1^ in all samples, and iron content varied from 38.2 to 126.5 µg g^−1^. Recommended daily allowance (RDA) for selenium for infants in the age of 0–6 months is equal to 12.5 µg/day. Assuming that infants consume about 750 mL of milk per day (c.a. 100 g of powdered milk), selenium RDA is provided [23]. In the case of Fe, daily intake significantly exceeds the RDA. For infants of 0–6 months, the RDA for Fe is 0.3 mg/day. Daily intake from pure 750 mL of milk is ca. 5 mg.

**Table 5:** Concentration levels of As, Cr, Fe and Se, X ± U

	As, ng g^−1^	Cr, ng g^−1^	Fe, µg g^−1^	Se, ng g^−1^
Cow milk-based formula 1	Nd	<100	57.9 ± 2.9	197 ± 6
Cow milk-based formula 2	Nd	<100	46.1 ± 3.7	130 ± 4
Cow milk-based formula 3	Nd	<100	70.1 ± 3.5	124 ± 4
Cow milk-based formula 4	Nd	<100	71.7 ± 3.6	132 ± 4
Rice porridge 1	47.8 ± 1.4	<100	57.0 ± 2.9	<5
Rice porridge 2	44.3 ± 1.3	<100	46.9 ± 3.8	<5
Rice porridge 3	46.2 ± 1.4	<100	46.9 ± 3.8	<5
Hypoallergenic formula 1	68.4 ± 2.1	<100	126.5 ± 6.3	<5
Hypoallergenic formula 2	72.2 ± 2.2	<100	91.2 ± 4.6	7.5 ± 0.4
Soya milk	50.6 ± 1.5	100 ± 15	64.1 ± 3.2	98.2 ± 3
Oatmeal porridge	15.2 ± 0.5	<100	38.2 ± 3.1	21.4 ± 0.6
Buckwheat porridge	<7	<100	99.6 ± 5.0	18.3 ± 0.6
Millet porridge	13.8 ± 0.5	<100	40.2 ± 3.2	10.0 ± 0.3
Spelt porridge	20.2 ± 0.6	<100	41.9 ± 3.4	16.7 ± 0.5

*Nd* Not determined

It should be noted that only producers of cow and soya-based milks give some information about trace elements concentration. In the case of porridges, only for rice-based products, information about iron and cooper content was given. For other porridges, there was not information about concentration of any elements except sodium. This could be a disturbing, taking into account, that this type of products (bio cereal porridge) are becoming more popular on the market, despite their relatively high price.

## Conclusions

The application of two analytical techniques with different physicochemical foundations ensures that accurate results were be obtained during investigation. It is especially important, when determining some problematic elements like As and Se. INAA method can be applied for the determination of Cr, Fe and Se when the concentrations of these elements are low. Determination of As below 100 ng g^−1^ is possible by NAA and above this concentration level also by ICP-MS method. Cr can be determined by ICP-MS method above 100 ng g^−1^ when DRC mode is applied. The expanded uncertainties of INAA U (k = 2) were estimated at 5–8 % for all elements, while in the case of ICP-MS method U (k = 2) were 15–20 %. The advantage of ICP-MS is shorter time of analysis comparing with INAA. The ratio primary reference measurement procedure based on RNAA is characterized by very low LOD. It allows to obtain results with lower uncertainty (U (k = 2) = 2–3 %) and shorten time of analysis comparing with INAA. As a rule, NAA method can be used to verify the correctness of the results obtained by ICP-MS method.

As, Cr, Fe and Se were determined in selected infant formulas by ICP-MS and NAA method. On the basis of the obtained results it can be concluded, that in cow milk-based formulas, independently of the producer and kind of milk, the content of trace elements is similar. The biggest differences in trace elements content are observed for porridges. It was find that daily intake of Se provided by analyzed infant formulas is sufficient but that of Fe is exceeded. Despite the large number of publications on the content of infant formulas, the further research should be continued—particularly in the relation to so called “bio”, “eco” products.
